# May CTC technologies promote better cancer management?

**DOI:** 10.1186/s13167-014-0023-x

**Published:** 2015-01-22

**Authors:** Martin Pesta, Vlastimil Kulda, Andrea Narsanska, Jakub Fichtl, Ondrej Topolcan

**Affiliations:** Department of Biology, The Faculty of Medicine in Pilsen, Charles University in Prague, Karlovarska 48, 301 66 Pilsen, Czech Republic; Biomedical Center, The Faculty of Medicine in Pilsen, Charles University in Prague, Pilsen, Czech Republic; Department of Biochemistry, The Faculty of Medicine in Pilsen, Charles University in Prague, Pilsen, Czech Republic; Department of Surgery, The Faculty of Medicine in Pilsen, Charles University in Prague, Pilsen, Czech Republic; Department of Internal Medicine II, The Faculty of Medicine in Pilsen, Charles University in Prague, Pilsen, Czech Republic

**Keywords:** Circulating tumor cells, Tumor markers, PPPM, Breast cancer, Colorectal cancer

## Abstract

In the case of cancer, death is usually not due to the primary tumor itself but due to dissemination. Analysis of the circulating tumor cells (CTCs), i.e., cells responsible for a formation of metastases, should provide information useful for the management of cancer patients, fulfilling the objectives of predictive, preventive, and personalized medicine (PPPM). Despite promising results, the decisions on stage of disease and how to guide the adjuvant treatment still do not include results of CTC assessment. We want to describe two major reasons why the recent diagnostic value of CTC analysis is not sufficient for clinical use. The first reason arises from the biological nature of the tumor itself and the second reason is associated with an interdisciplinary status of CTC diagnostics in the sense that it is neither a theme purely for pathologists nor for haemato-oncologists nor clinical biochemists. We anticipate that there are at least three areas where CTCs can be useful for clinical practice. The first is monitoring of treatment efficacy of cancer patients. The second is a molecular characterization of captured CTCs for targeted treatment, and the third is a cultivation of captured CTCs for drug sensitivity testing. All of these approaches allow researchers recognize and respond to changes of phenotype of cancer cells during disease progression and introduce PPPM into clinical practice.

## Review

### Introduction

In the case of cancer, death is usually not due to the primary tumor itself but due to dissemination, i.e., the formation of distant metastases, that may develop years after the removal of the primary tumor. Even though no evidence of tumor spread may be seen at the time of the primary diagnosis, as we can see in breast cancer, a relevant number of axillary lymph node-negative breast cancer patients also develop local or distant metastases [1,2]. Although imaging has been, and still is, the gold standard for prognosis estimation and disease monitoring, there are emerging alternative approaches that could reveal micrometastases earlier and, combined with traditional methods, could improve monitoring of the disease status [3]. Monitoring and analysis of the cells responsible for metastases formation should give information useful for the treatment of cancer patients and serve in the future as a real-time “liquid biopsy” [4].

The presence of detached tumor cells in peripheral blood, similar to cells of the primary solid tumor, was first recognized and described by Thomas R. Ashworth in 1869 [5]. The intensive research in last 15 years has brought progress in knowledge on how the tumor is spread by tumor cells in the peripheral blood, lymph nodes, or bone marrow and about the phenotype and genotype of these cells. We are witnessing a paradoxical situation. This deeper knowledge shows the complexity and nonuniformity of the metastatic process, resulting in delays of the use of circulating tumor cells (CTCs) in the clinical management of oncological patients on the one hand, while on the other hand, allowing individualization of treatment in the future.

The goal of this review is to summarize problematic points that may postpone implementation of CTC assessment in clinical practice, i.e., issues that are currently being dealt with in relation to the clinical value of CTCs. We also want to discuss in which areas of cancer management of patients there is the greatest benefit of CTC examinations. For this, we have specifically focused on CTC assessment in breast and colorectal cancer.

We want to describe two major reasons why the recent diagnostic value of CTC analysis is still not sufficient for routine clinical use. The first reason arises from the biological nature of the tumor itself, for example, intratumoral heterogeneity and its relation to the composition of the population of cells detached into the lymphatic system and blood stream. Understanding the mechanisms behind the epithelial-mesenchymal transition (EMT) and the transformation from cancer stem cells to differentiated cells helps to reveal properties of subpopulation of CTCs. The second reason is associated with an interdisciplinary status of CTC diagnostics in the sense that it is neither a theme purely for pathologists nor for haemato-oncologists nor clinical biochemists. Despite whether the origin of CTCs is in the primary tumor or its metastasis, i.e., in solid tumor, the place of detection is the blood. Therefore, this problematic issue is not in the scope of pathologists. As the origin of CTCs is not in haematopoetic cells, it is not a field of interest for haemato-oncologists. It is possible to say that this marker becomes a topic for biochemists as a potential oncomarker. One of the consequences of this interdisciplinary character of CTCs is also a broad spectrum of detection techniques currently used in research. There are methods similar to pathology methodology which utilizes microscopy (HD-CTC assay technology licensed to Epic Sciences, San Diego, CA, USA) [6], while the other techniques are similar to flow cytometry (CellSearch system licensed to Veridex, Raritan, NJ, USA) [7], or the methods are based on a combination of immunomagnetic enrichment and molecular biology approach (RT-PCR) [8]. However, we can also find the creative methods such as photoacoustic flow cytometry [9] or microfluidic platforms [10]. The broad spectrum of methods associated with the detection of CTCs is based on their various phenotypical properties, and so, it is difficult to compare data obtained by different techniques [11]. This therefore results in a lack of large randomized trials that would confirm or refute the benefits of patients’ management, based on decisions made by the determination of CTCs.

### Definition of CTCs and metastatic phenotype of tumor cells

The major challenge of the current research of CTCs is to clearly define the phenotype of cells that are the source of new tumor nests (metastasis). As such, this question is tightly coupled with understanding carcinogenesis as a whole. To date, the significance of the role of cancer stem cell phenotypes in primary tumor growth and the role of EMT in cancer progression is still not fully understood [12–14].

Simply, a CTC is a tumor cell present in the peripheral blood, as a general definition. However, presently, there is no generally accepted agreement about CTC phenotypes—i.e., surface markers defining CTC. This is one of the reasons that there are many approaches for detection of CTCs. Most detection systems use markers of CTCs epithelial cell adhesion molecule (EpCAM) and cytokeratins (intermediate filaments specific for epithelial cells). EpCAM is expressed to various degrees by many epithelial tumor cells and functions as a homotypic calcium-independent cell adhesion molecule [15,16]. Cytokeratins are broadly expressed by epithelial tumor cells, including both low (8, 18, and 19) and high-molecular weight (1, 5, 10, and 14) cytokeratins [17]. Both EpCAM and cytokeratins are detectable by immunofluorescence techniques.

**Figure 1:**
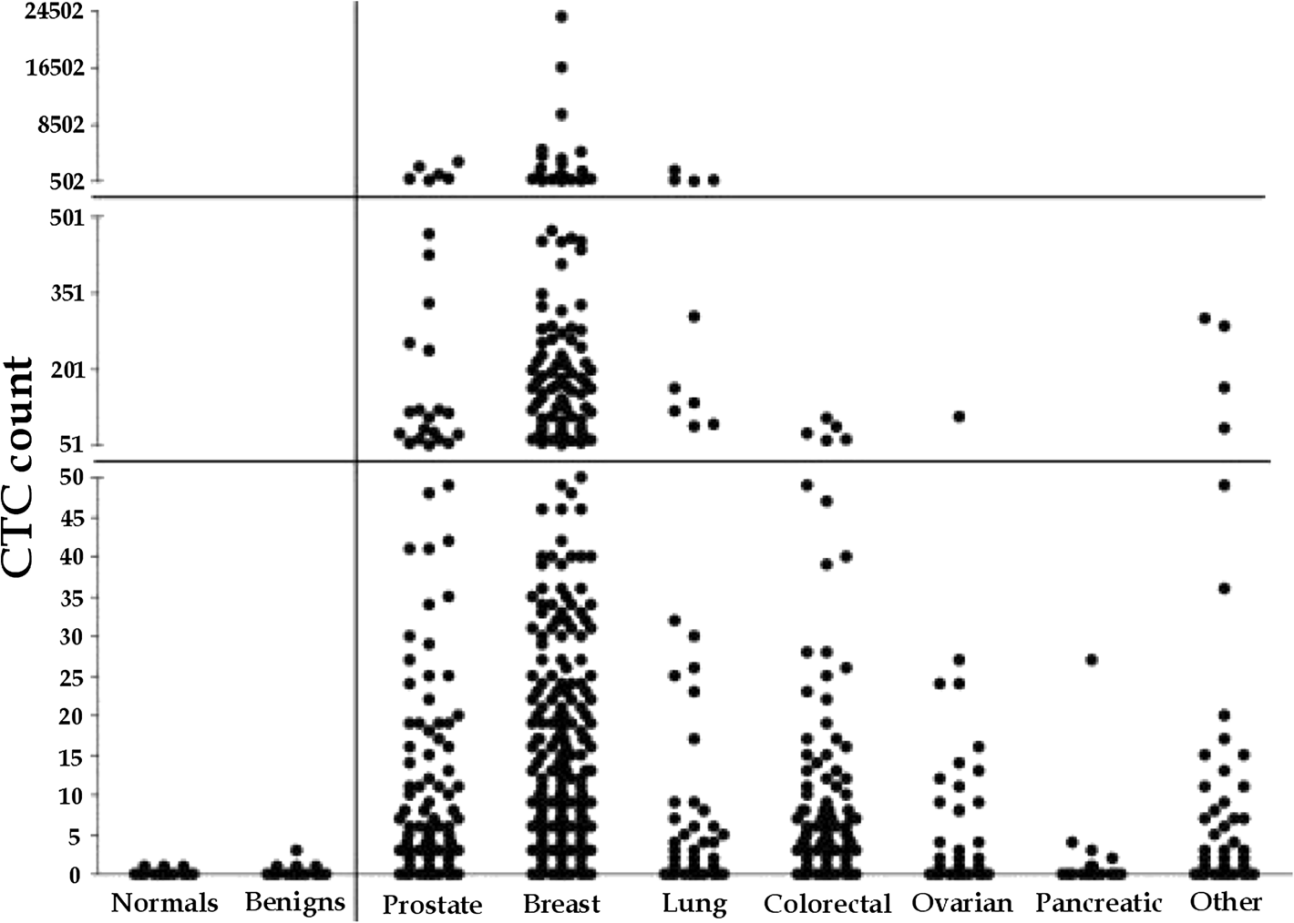
**CTC count in different metastatic cancer types; data adapted from [** 18 **].** The plot compares CTC counts enumerated by CellSearch system (the number of CTCs in 7.5 ml of whole blood) from healthy donors (normals) and patients with nonmalignant diseases (benigns) with CTC counts from patients with metastatic prostate, breast, lung, ovarian, colorectal, pancreatic, and other cancers.

A CTC is identified by many methods as a nucleated cell that is positive for EpCAM, is cytokeratin positive, and is CD45 negative. This strategy targets tumor cells in peripheral blood as cells having a nonhematopoetic phenotype with epithelial characteristics. On the basis of this CTC definition, a lot of successful work has been done. For example, it was found that the amount of CTCs differs in various types of tumors, as was shown in the work of Allard et al., Figure 1, [18]. In some groups of patients, it was found that the number of CTCs detected is related to prognosis [19–21]. The simplest approach, enumeration, is based on the number of detected CTCs. The result (the number of detected CTCs) strongly depends on detection technique. It is necessary to mention that this approach passes other properties and processes which are to current knowledge associated with malignant potential as EMT or cohesive and collective cell migration.

Despite many clinical studies that have confirmed the prognostic potential of EpCAM positive CTCs, there is evidence that cannot be ignored and that the cells responsible for formation of metastases can be those which express stem cell properties and also express some molecules typical for cells that have undergone EMT [22,23]. Such cells do not necessarily express EpCAM and may be missed by currently used detection techniques [24,25]. Moreover, recent findings have shown that CTCs are not a uniform population [26] and it is apparent that not all of them have the same potential to form secondary tumors.

From a statistical point of view, it is not surprising that an adverse outcome is predicted by a greater number of CTCs detected in peripheral blood on the basis of EpCAM+, CK+, and CD45- phenotype. Despite the fact that the specific CTCs that are detected may not have metastatic potential themselves, it can be expected that the detection of a higher number of these CTCs predicts an increase in those CTCs with metastatic potential. Therefore, although we do not detect the right CTCs, we probably obtain the correct result.

The majority of studies targeted for detection of CTC CK+ and EpCAM+ phenotype and deal with breast and colorectal cancer patients. Therefore, in this paper, we focused on CTC assessment in these tumor types.

#### CTCs as prognostic markers in breast cancer

Breast cancer is the most commonly diagnosed type of cancer in women, and the risk of development of this disease during life is one in nine [27]. Primary detection and diagnosis is mainly based on imaging methods such as ultrasound and mammography [28]. Breast cancer is a highly heterogeneous disease, and the histopathological classification includes some 20 major tumor types and 18 minor subtypes [29]. For the treatment, basic classification is based on the assessment of expression of estrogen receptors (ER), progesterone receptors (PgR), and HER2 status [30]. Imaging methods alone cannot provide information about the definite tumor type [31], and the possibility of monitoring of disease development is also limited, especially by size of tumor mass.

The role of CTCs as prognostic markers has been reported by many clinical studies, and several clinical trials in both, primary and metastatic breast cancer [32–36], all supporting a significant correlation between CTC amount, progression-free survival (PFS), and overall survival (OS). This correlation was recently confirmed by a meta-analysis involving data of 1,944 patients from 17 European centers, with the conclusion that CTC count is an independent prognostic marker of PFS and OS [37].

Until recently, most of the large studies have been done on a group of patients with metastatic breast cancer. This year, results of the SUCCESS study, a large patient cohort study focused on early breast cancer, were published. This trial provides strong evidence for the prognostic relevance of CTCs both before and after adjuvant chemotherapy and supports the clinical potential of CTCs to assess the individual risk of patients at the time of primary diagnosis [38]. The prognostic role of CTC enumeration has been shown to be dependent on the type of primary tumor and is more pronounced in ER-positive and triple-negative than in HER2-positive metastatic breast cancer [39].

#### CTCs as prognostic markers in colorectal cancer

Colorectal cancer (CRC) is another leading cause of cancer-related death in developed countries [40]. Five year survival rates are over 90% for stage I disease and are lower than 10% for patients in stage IV [41]. However, up to 25% of the patients with localized disease subsequently develop disease relapse and metastases [42]. Major effort is to improve the survival rates for patients with metastatic CRC. Targeted therapies, based on the use of monoclonal antibodies directed against the epidermal growth-factor receptor (EGFR) and vascular endothelial growth factor (VEGF), have been shown as promising treatments [43].

The role of CTCs as prognostic markers for primary colorectal cancer has been reported in many studies [44,45], that generally concluded that the presence of CTC in peripheral blood is a marker of poor disease-free survival in patients with nonmetastastic CRC. Similarly, detection of CTCs in peripheral blood of patients with resectable colorectal liver metastases or widespread metastatic CRC is associated with disease progression and poor survival. In multivariable analyses, the detection of CTCs is an independent prognostic factor [46].

Despite these promising results, the decisions on stage of disease and how to guide the adjuvant treatment (oncological guidelines) still do not include the results of CTC assessment. It could be expected that the implementation to standards of care could be facilitated by a standardized and automated system for CTC detection, e.g., CellSearch, which currently holds a dominant position in the field of CTC detection instruments.

The complexity of the CTC topic becomes apparent in comparison with immediate integration of KRAS mutation assessment into routine oncological clinical practice. The analysis of the presence of KRAS mutations in tumor tissue in patients treated with EGFR-tyrosine kinase inhibitors (TKIs) was conducted in works of Pao et al. [47] and Massarelli et al. [48]. These studies suggested an association between KRAS mutations and an absence of response to EGFR-TKIs treatment. Since 2009, KRAS mutational status has been recommended to guide the therapy in oncological patients treated by EGFR-TKIs and anti-EGFR monoclonal antibodies, according to KRAS mutational status. This was the first genetic test to guide the treatment of cancer [49–51].

### CTCs in management of oncological patients

Nevertheless, there is still a question regarding the place for CTC assessment in clinical oncology and if this place exists at all. It remains to be determined whether an alternative approach, which is easier and cheaper, could provide similar information. From our point of view, there are at least three areas where CTCs can be useful for clinical practice. First is the monitoring of treatment efficacy of cancer patients. Second is the molecular characterization of captured CTCs for targeted treatment, and third is the cultivation of captured CTC for drug sensitivity testing. All these assessments provide information about the current status of the disease and allow a personalized approach.

### Monitoring of therapy

In clinical practice, imaging technologies such as computed tomography (CT), positron emission tomography (PET), and magnetic resonance imaging (MRI) and laboratory determination of tumor marker levels are well-established methods for monitoring cancer patients and determining the effect of treatment. However, using these approaches, long periods are required to record reliable information on the effect of the treatment, and during this period, the patients can be exposed to potentially unnecessary therapy that may increase treatment-associated complications, such as neutropenia, neuropathy, and alopecia. Monitoring of CTC level has an inherent potential to provide information on the effects of treatment. The level of CTCs appears to respond more rapidly to the treatment, in as little as a few weeks [52,53].

In 2006, Budd et al. did a direct comparison between enumeration of CTCs and radiological imaging in metastatic breast cancer patients for prediction of OS [54]. One hundred and thirty-eight metastatic breast cancer patients had imaging studies done before and a median of 10 weeks after the initiation of therapy. CTC counts were determined 4 weeks after initiation of therapy. This study showed for the first time that CTC enumeration is a reliable and accurate way to monitor disease progression, offering an earlier and more reproducible monitoring than standard anatomic imaging methods [54].

Liu et al. showed that five or more CTCs predict for poorer PFS in patients with metastatic breast cancer and demonstrated a strong correlation between CTC results and radiographic disease progression monitoring in patients receiving chemotherapy or endocrine therapy. Importantly, correlation was applied to CTC results obtained at the time of imaging before imaging and after imaging [55]. Similar results to Liu et al’s study have also been published. For example, a study by De Giorgi showed the possibility that the number of detected CTCs, might be connected to the loci of metastasis, and that a higher number of CTCs might indicate bone metastasis [56,57].

In 2014, a study “Circulating Tumor Cells and Response to Chemotherapy in Metastatic Breast Cancer: SWOG S0500” concluded with a disappointing outcome. Despite this study confirming the prognostic significance of CTCs in patients with metastatic breast cancer receiving first-line chemotherapy, for patients with persistently increased CTCs after 21 days of first-line chemotherapy, early switching to an alternate cytotoxic therapy was not effective in prolonging OS. For such patients, there is a need for a more effective treatment than standard chemotherapy. It is obvious that monitoring of the effect of cancer treatment is particularly important when we can offer to patients another effective treatment [58]. This result showed that successful use of CTC in patients’ management, especially in monitoring of therapy, is coupled with the availability of alternative effective drugs and other lines of treatment.

### Molecular characterization of captured CTCs for decisions about the treatment

Molecular characterization of CTCs at the DNA level and assessments of expression at the RNA and protein levels can provide information on the presence of therapeutic targets and also the presence of changes that are predictors of ineffective therapy. For the patients and their clinicians, the question of properly selected treatment is absolutely crucial. As well as analysis of the tumor tissue itself, analysis of CTCs might provide information about the current molecular targets for treatment and predictors of resistance. As it has been shown, the results from both sources are not always consistent [59–61]. In the time of progression and recurrence of disease, genotype and phenotype are changing and re-evaluation of current molecular targets is a strategy with clinically exploitable potential.

Finding molecular targets and correctly defining target groups of patients is important to pharmaceutical companies; molecular characterization of CTCs may contribute to the development of novel anticancer drugs.

#### Molecular characterization of CTCs in breast cancer

In breast cancer, the presence of HER2/neu receptor on the surface of CTCs could be useful molecular characteristic. Knowledge of the HER2/neu receptor expression, not only in tumor cells of the primary tumor but also in cells released from it, CTCs, can help determine the correct choice of treatment for patients with breast cancer. A study conducted by Wülfing et al. showed that HER2-positive CTCs indicated poor clinical outcome in stages I to III breast cancer patients. In addition, Wülfing observed in 12 (from 17) patients with HER2-positive CTCs, the primary tumor was negative for HER2, as assessed by immunohistochemical score and fluorescence *in situ* hybridization [62]. Similarly, in another study, Munzone et al. recorded 18% discordance between HER2-positive CTCs and HER2 status of the primary tumor cells [60]. There is also evidence that HER2 status can change during disease recurrence or progression in breast cancer patients. Fehm et al. provide HER2 status of CTCs in patients with metastatic breast cancer and observed HER2-positive CTCs in 32% and 49% (CellSearch assay and AdnaTest BreastCancer, respectively) of patients with HER2-negative primary tumors [63]. In our study on early breast cancer patients as luminal A type, we also observed a discrepancy between HER2 status of the primary tumor cells and HER2-positive CTC (unpublished data).

Discrepancies between the molecular characteristics of the primary tumor and CTCs are targets of interest in a randomized phase II trial for patients with HER2-negative primary breast cancer, who after completing (neo)adjuvant chemotherapy and surgery, have detectable CTCs in their peripheral blood. Nonmetastatic HER2-negative patients, but with detectable CTCs by CellSearch after surgery in their peripheral blood and HER2/neu-positive CTCs, were randomized after neoadjuvant treatment to receive adjuvant trastuzumab [64]^a^. The study is coordinated by European Organisation for Research and Treatment of Cancer, Network of Core Institutions, and the results will be available in 2015.

At the time when metastatic disease is identified, knowledge of HER2/neu status could help to optimize treatment decisions. Since HER2 positivity could be acquired during disease progression, assessment of the HER2 status in CTCs may be clinical important in patients whose HER2 status was not determined in the primary tumor. This is especially relevant in patients where tissue sampling and direct analysis of metastatic tissue may be difficult to obtain (due to its location). In a study by Fehm, a subgroup of patients with initially negative or unknown HER2 status can have HER2-positive CTCs at the time of development of metastatic disease. Fehm observed that eight out of 21 breast cancer patients with detectable CTCs and with negative or unknown primary tumor HER2 status exhibited HER2 amplification [59].

#### Molecular characterization of CTCs in colorectal cancer

Molecular characterization of CTC may prove to be useful in the management of patients with metastatic CRC by helping to predict treatment response to biological therapy. In patients with metastatic CRC prior to administering therapy that uses anti-EGFR monoclonal antibody, the test of the presence of activating KRAS mutation is recommended. KRAS and BRAF mutations are currently assessed in the primary tumor tissue. It was shown that the mutation status of the primary tumor is not always identical with metastases. Santini et al. [65] and Molinari et al. [66] performed analysis of KRAS mutations from primary CRC and from visceral and lymph node metastasis, finding a concordance of 96% and 92%, respectively. Baldus et al. [67] observed a concordance rate between primary tumor and visceral metastasis (90%), but not with lymph nodes metastasis (31%). On the other hand, Tortola et al. [68] found a significant discordance rate between tumor and related bone marrow metastases.

It is not suppressing that not all patients with wild type KRAS in the primary tumor are successfully treated with anti-EGFR antibodies. In the case of doubts of KRAS status in the primary tumor, tests of KRAS mutations in metastatic tissue could be useful, but this can be limited by obtaining patients’ cancer tissues. To improve patient selection, assessment of mutation status in CTCs could possibly better represent the presence of mutations in metastases, than the primary tumor. As shown in the work by Yen et al., the detection sensitivity, specificity, and accuracy of membrane-arrays for CTCs with KRAS oncogene significantly correlate to KRAS mutations in tumors (*P* < 0.0001) [69]. Other studies have shown similar results, but it is necessary to mention that different methods of CTC detection were used [70]. Sastre’s *post hoc* analysis showed that CTC count and their KRAS status were independent prognostic factors for outcomes in patients with metastatic CRC treated with bevacizumab ± chemotherapy [71]. Similarly, Kuboki’s study showed that a high CTC count predicted reduced OS in patients with advanced CRC treated with cetuximab-combination chemotherapy as a third-line treatment [72].

These results suggest that the assessment of CTCs might provide important predictive and prognostic information for such patients and that the inclusion of KRAS status of CTCs into other trials certainly makes sense. However, the reasons of unsuccessful treatment are complicated. The work of Gasch et al. points to considerable intra- and interpatient heterogeneity of EGFR expression and genetic alterations in EGFR, KRAS, and PIK3CA, thus possibly explaining the variable response rates to EGFR inhibition in patients with CRC [73].

As well as CTC KRAS status determination, assessment of other genes in captured CTC may improve prediction of treatment response. Determining the expression of genes of tumor cells involved in the metabolism of chemotherapeutic agents also can predict the effect of this treatment. Gazzaniga et al. [74] determined the expression profile of multidrug resistance-related proteins (MRPs) of 105 patients with diagnosis of carcinoma, in CTCs isolated from peripheral blood by CellSearch. Authors were able to identify a drug-resistance profile of CTCs, which is predictive of response to chemotherapy, independent of tumor type and stage of disease with sensitivity 98% and specificity 100% [74].

### Chemosensitivity testing of circulating tumor cells *in vitro*

To destroy the tumor cells remaining after surgery, adjuvant chemotherapy is applied to eliminate disseminated cells. Today, therapy for a particular patient is applied on the basis of TNM classification and guidelines based on clinical trials using statistically evaluated longest relapse-free survival for a given combination of treatment. Unfortunately, this does not guarantee whether the chosen treatment will be successful in individual patients. It is obvious that the testing of sensitivity to therapeutic agents directly to the tumor cells of a particular patient would help to determine appropriate treatment. One way, not always possible, is a cultivation of tumor cells from the primary tumor. However, tumors consist of heterogeneous cell populations [75–77], and it is not clear which cell subpopulation will finally be able to form metastases [78]. The cells released from the tumor tissue in the peripheral circulation are those that are responsible for a formation of metastases, and their analysis can help to choose the best treatment option.

To obtain CTCs, a number of methods may be used, that can be generally divided into two groups. There are methods of isolating cells on the basis of phenotypic properties (surface antigens) which means that it passes all other cells, however, among which may be even those which have tumor character. In contrast, there are methods using all blood cells (red blood cell lysis and one centrifugation step). This approach avoids preanalytic manipulation with the samples. Circulating epithelial cells obtained by these methods could be used to test drug sensitivity, not only in the metastatic patients but also after primary tumor resection before adjuvant therapy. Circulating epithelial cells have been shown to respond to therapy in the same way as the primary tumor [79], and, therefore, it seems appropriate to test the actual sensitivity of the residual tumor mass to chemotherapeutic treatment. A study by Rudiger et al. showed that chemosensitivity testing of CTC provides real-time information about the sensitivity of the tumor present in the patient, even at different times during therapy, and correlates with treatment success. For isolation of CTC of breast cancer and ovarian cancer patients, this author used red blood cell lysis and one centrifugation step and the cell suspension was subsequently incubated under cell culture conditions with the drugs in question [78]. Gallant et al. in their work for isolation of CTCs from peripheral blood of CRC patients used a microfluidic device that enriches CTCs by size and deformability and showed that isolating a low number of viable CTCs and maintaining them in a culture for a few weeks is possible [80].

Drug sensitivity testing on CTCs is a step to personalized cancer treatment strategy, i.e., individualization of chemotherapy in cancer patients on the base of actual state of disease. Despite the above discussion, it should be noted that there remain many questions not fully answered. To what extent it is possible to achieve reproducibility of assessments (in comparison to traditional tumor markers) in one such question. Of course, there will be limitations as a result of the kinetics of CTCs in the blood stream. Finally, it must be mentioned that there are other techniques which we can use to obtain similar information that can be brought by CTCs to oncological patients. Promising approaches include free circulating nucleic acids in plasma or serum (CNAPS). This approach uses the extracellular tumor-derived DNA and/or RNA for detection of tumor properties [81,82]. Such obtained nucleic acids can undergo the same molecular genetic analyses as captured CTCs. On the other hand, such approaches do not allow morphological analysis of tumor cells.

## Conclusions

Before we are able to use real personalized diagnostics and treatment, it will be necessary to deeply understand tumor pathogenesis and further phenotype of metastatic cells and processes they undergo, so that treatment decisions made in individual patients were on the basis of causal relation of cell phenotype to disease development. If we suppose that effective treatment of patients with cancer will be based on targeted therapy directed against changes of DNA in tumor cells, we must be able to analyze tumor cells (DNA from tumor cells), moreover, repeatedly during the disease course. Chemosensitivity testing on captured CTCs provides direct information on the effect of the proposed therapy. These are the areas of cancer management where CTCs are most useful and promising.

The main benefit for the patient is that CTC assessment is a noninvasive and repeatable tool for individualization of treatment. It should help not only in primary setting of the treatment but also in verification of previous treatment decisions and adjustment of treatment according to current changes.

CTC analysis could be a part of integrative medical approach of the multimodal diagnostics, disease-specific biomarker patterns, individual patient profiles, and treatments tailored to the person [83] which are the objectives of PPPM.

Use of the diagnostic potential of CTCs for cancer patient treatment requires the philosophy and innovative paradigm as published in the EPMA White Paper [84].

## Endnotes

^a^The study is coordinated by European Organisation for Research and Treatment of Cancer, Network of Core Institutions, and the results will be available in 2015.

## Abbreviations

CEA: Carcinoembryonic antigen
CRC: Colorectal carcinoma
CTCs: Circulating tumor cells
EGFR: Epithelial growth factor receptor
EMT: Epithelial-mesenchymal transition
EpCAM: Epithelial cell adhesion molecule
PPPM: Predictive preventive, and personalized medicine
RT-PCR: Reverse transcription polymerase chain reaction
VEGF: Vascular endothelial growth factor
